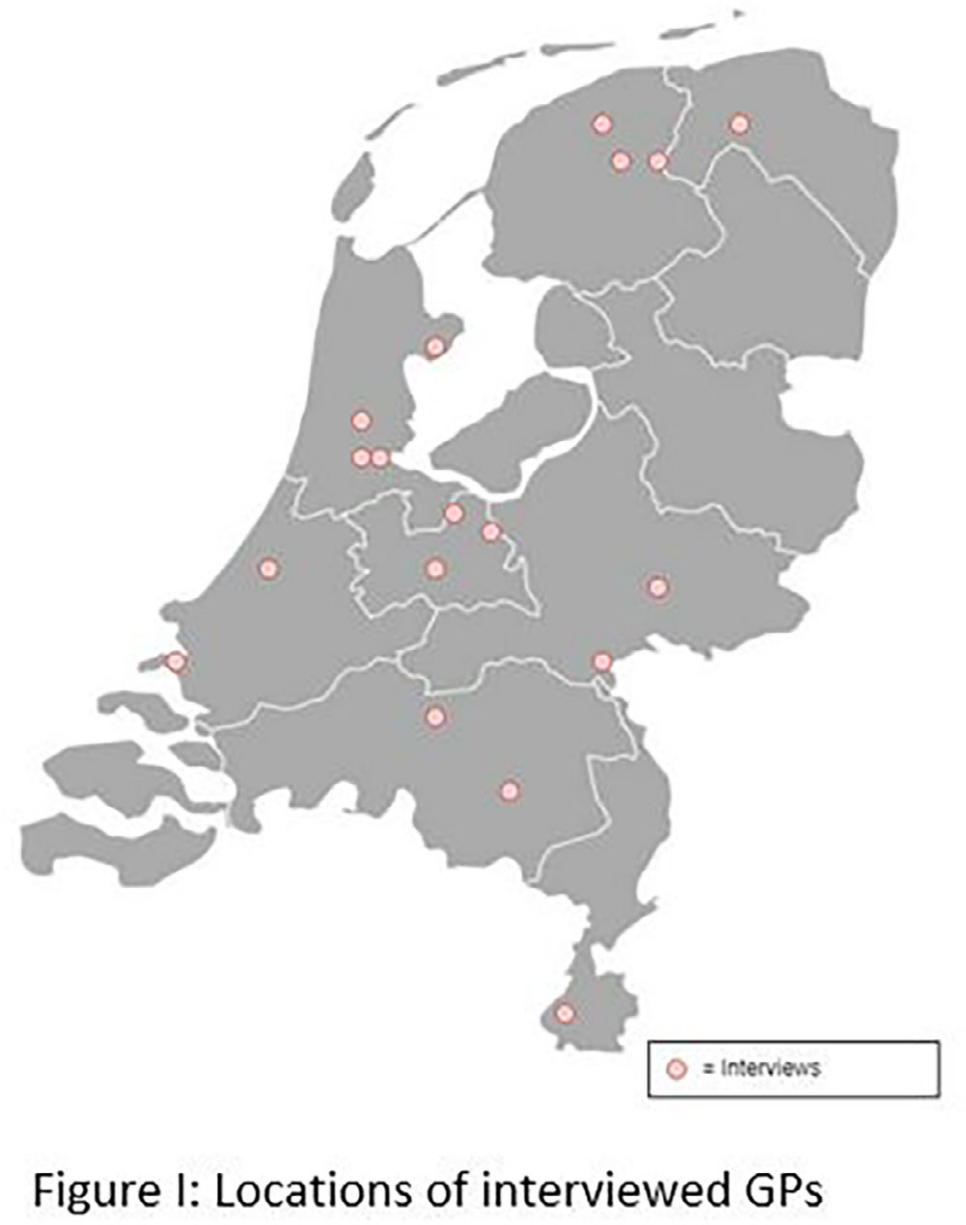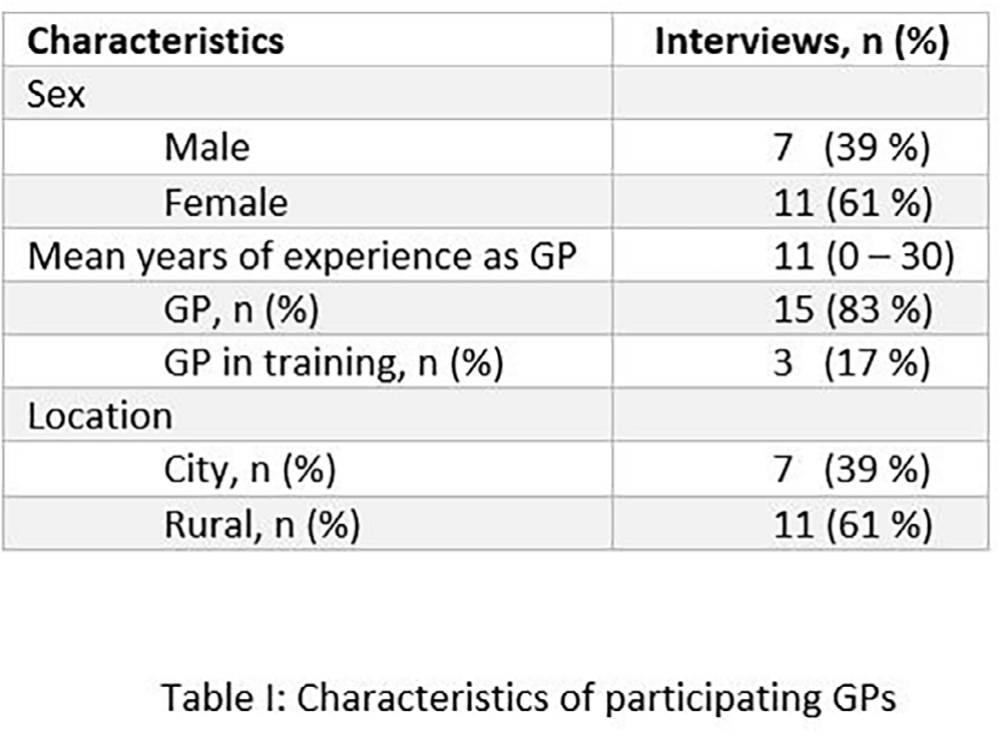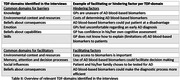# Exploring barriers and facilitators for the implementation of Alzheimer’s disease blood‐based biomarkers in primary care: The CANTATE‐Perspectives study

**DOI:** 10.1002/alz.088783

**Published:** 2025-01-09

**Authors:** Thomas Claessen, Fleur C.W. Visser, Barbara C. van Munster, Wiesje M. van der Flier, Martí Jiménez‐Mausbach, Argonde C. van Harten, Leonie N.C. Visser, Charlotte Teunissen

**Affiliations:** ^1^ Alzheimer Center Amsterdam, Department of Neurology, Vrije Universiteit Amsterdam, Amsterdam UMC, location VUmc, Amsterdam Netherlands; ^2^ Amsterdam Neuroscience, Neurodegeneration, Amsterdam Netherlands; ^3^ Neurochemistry Laboratory, Department of Clinical Chemistry, Amsterdam Neuroscience, Vrije Universiteit Amsterdam, Amsterdam UMC, Amsterdam Netherlands; ^4^ University of Groningen, University Medical Center Groningen, Groningen Netherlands; ^5^ Alzheimer Center Groningen, Groningen Netherlands; ^6^ Alzheimer Center Amsterdam, Neurology, Vrije Universiteit Amsterdam, Amsterdam UMC, Amsterdam Netherlands; ^7^ Novo Nordisk A/S, Copenhagen Denmark; ^8^ Amsterdam Public Health, Quality of Care, Personalized Medicine, Amsterdam Netherlands; ^9^ Department of Medical Psychology, Amsterdam Public Health research Institute, Amsterdam UMC, location AMC, Amsterdam Netherlands; ^10^ Division of Clinical Geriatrics, Center for Alzheimer Research, Department of Neurobiology, Care Sciences and Society, Karolinska Institutet, Stockholm Sweden; ^11^ Alzheimer Center Amsterdam, Neurology, Vrije Universiteit Amsterdam, Amsterdam UMC location VUmc, Amsterdam Netherlands

## Abstract

**Background:**

Adequately diagnosing Alzheimer’s disease (AD) in primary care can be challenging. Early symptoms often go unrecognized and other (neurodegenerative) diseases may be misdiagnosed as AD. AD blood‐based biomarkers could improve the diagnostic process for cognitive complaints in primary care. To prepare for implementation of these biomarkers in different contexts, we investigated barriers and facilitators from the perspective of general practitioners (GPs).

**Method:**

In this qualitative, semi‐structured interview study, we used purposive sampling to select a heterogeneous group of 18 Dutch GPs (Figure I & Table I). The interview guide was based on the “Theoretical Domains Framework (TDF). Interviews were audio‐recorded and transcripts analyzed using MaxQDA software. By means of directed content analysis, a multidisciplinary research team (TC, FV & LV) identified barriers and facilitators for implementation of AD blood‐based biomarkers in primary care and categorized these into TDF‐domains.

**Result:**

We identified 8 relevant TDF‐domains (Table II). Barriers could be mapped on the environmental context and resources (e.g. AD blood‐based biomarkers not being in GP guidelines) and knowledge domains (e.g. GPs perceived AD‐dementia as a syndromic diagnosis, not a biological diagnosis and were unaware of AD blood‐based biomarkers). Other important domains were emotion and beliefs about consequences (e.g. AD blood‐based biomarkers could result in over‐ or underdiagnosis of AD).

Facilitators could be mapped onto the environmental context and resources domain (e.g. availability of (disease‐modifying) treatment for AD, availability of education and accessible information for GPs and patients). Furthermore, facilitators could be mapped onto the social influences, beliefs about consequences & memory, attention and decision processes domains (e.g. GPs believed that AD blood‐based biomarkers would be useful in primary care as biomarkers could initiate further care, facilitate decision‐making and provide clarity regarding the diagnosis).

**Conclusion:**

We identified a large variety of barriers and facilitators that can help to further understand and enable the implementation of AD blood‐based biomarkers in primary care. Important barriers and facilitators include the need for education and accessible information to enhance GPs’ knowledge about AD blood‐based biomarkers. Our next step is to further explore and confirm relevant factors for successful implementation by means of focus groups and a large scale questionnaire.